# Black rice addition prompted the beer quality by the extrusion as pretreatment

**DOI:** 10.1002/fsn3.1223

**Published:** 2019-09-27

**Authors:** Tianyu Zhang, Haijing Zhang, Zhe Yang, Yiran Wang, Hongjun Li

**Affiliations:** ^1^ School of Agricultural Engineering and Food Science Shandong University of Technology Zibo China; ^2^ Shandong Drug and Food Vocational College Zibo China

**Keywords:** amino acid, extruded black rice adjunct beer, HS‐SPME‐GC–MS, nitrogenous substances

## Abstract

Flavor compounds, including total polyphenols, amino acids, and protein, in beer with extruded black rice as adjunct were detected and analyzed. Beer brewing technique has been intensively investigated in the past century. The chase of beer quality, including the color, flavor, foam, nutrition, and functionality, attracted considerable attention. Hence, headspace solid‐phase microextraction in combination with a gas chromatography coupled to mass spectrometry (HS‐SPME‐GC–MS) was used to analyze flavor compounds qualitatively and quantitatively. A total of one organic acid, one aromatic, ten alcohols and 23 esters were present in extruded black rice adjunct beer. Protein components and molecular weight were analyzed, and the results were consistent with those of traditional beer in terms of content of foam‐stabilizing protein. The contents of essential amino acid which is an important nutritive index were higher than those in traditional rice adjunct beer, especially valine (70.9 mg/L) and threonine (42.8 mg/L). The representative ingredients of extruded black rice adjunct beer were polyphenols, nerolidol, geraniol, and geranylgeraniol which affected the functionality and antioxidant ability.

## 1 INTRODUCTION

Beer is the second most consumed (accounting for 37%) alcoholic beverage in Europe, with the average per capita consumption of 72.8 L in Europe in 2009–2011, according to the European Spirits Organization (Quiferrada et al., 2015). At present, up to 85%–90% of beer in the world is produced with adjuncts (Mallawarachchi, Bandara, Dilshan, Ariyadasa, & Gunawardena, 2016). Indigenous cereals are generally used as adjuncts, thereby supporting local agriculture. Sorghum is the most common adjunction in Africa, while it was rice in Asia, corn in America, and barley and corn in Europe (Mallawarachchi et al., 2016).

Extrusion processing is used widely for the improvement or modification of the food material qualities (Dale, Young, & Makinde, 2013; Zhang, He, Cao, Ma, & Li, 2017). During extrusion, starch gelatinization is performed at higher pressures and temperatures and also contributes to the breakdown of starch by mechanical shear force (Singh & Smith, 1997). This method used in raw material pretreatment can shorten the production cycle and increase the using rate of material (Dale et al., 2013).

The use of special rice malts can enhance the flavor, taste, color, and body of rice malt beer (Ceccaroni et al., 2019). Black rice which is a special type of rice with color pigments has been widely cultivated and consumed in Southeast Asian countries for decades because black rice is characterized by the high content of polyphenols, particularly anthocyanins (Jin, 2010), other antioxidant compounds, such as flavones, proanthocyanidins, and phenolic acids which contribute to its healthy nutritional profile (Abdel‐Aal, Christopher, & Iwona, 2006). Black rice has beneficial effects on human health and reduces the risk of developing chronic disease (Dipti et al., 2012; Samyor, Das, & Deka, 2017). A review has shown that headspace solid‐phase microextraction coupled with gas chromatography–mass spectrometry (HS‐SPME‐GC–MS) has been proved as an important tool for the rapid, accurate analysis of liquid foods in many instances (Tian, 2010). Headspace solid‐phase microextraction coupled with gas chromatography–mass spectrometry is a well‐established procedure for volatile flavor analysis in beverages (da Silva et al., 2015).

Nitrogenous compounds which exist in nucleic, proteins, polypeptides amino acids, and biogenic amine are extremely important in the aspect of beer nutrition and stability of the finished product (Marta Fontana, 2009). These compounds are in endless variety in different beers due to different materials and processing. Some specific amino acids and proteins are reported as off‐flavors and result in instability of product; in combination with other beer compounds, these factors contribute to the taste and drinkability of beer (Poveda, 2019).

To the best of our knowledge, no research has been performed on the use of extruded black rice as adjunct for specialty malt production. In addition to the stability and the nutritional value of the beer, the use of the black rice malts enhanced the flavor and color due to their high polyphenol content and good antioxidant capacity which can attract new customers.

## 2 MATERIALS AND METHODS

### 2.1 Materials and chemicals

Australian malt was obtained from Jinan Shuangmai Beer Supplies Co., Ltd.Rice, and black rice was acquired from a local market in Zibo, Shandong Province, China. Ethanol, acetaldehyde, n‐propyl alcohol, n‐butanol, isobutanol, isoamyl alcohol, isoamyl acetate, ethyl acetate, and ethyl caprylate were of chromatographic grade; ethyl acetate was of analytical grade. All chemicals were produced from Tianjin Shield Fine Chemicals Co., Ltd.

### 2.2 Raw material processing

Rice and black rice were adjusted to 21% moisture level and extruded using a homemade extruder with automatic control system. The extruder possessed a 79 mm barrel bore, 16.4:1 length‐to‐diameter ratio, 77 mm outer diameter screw and 8 mm diameter bore. Feed rate (100 kg/h), screw speed (200 r/min), and extrusion temperature (70°C) were kept constant in all experiments.

After extrusion, the materials were allowed to cool at room temperature, and then, we adjusted the moisture to 13% and reduced the particle size to 0.9 mm by crushing. The malt was crushed by a crusher.

### 2.3 Beer production

The extruded black rice flour was homogeneous mixture with crushed malt. Black rice flour (6.80 kg) and malt (15.20 kg) were poured into a 150 L saccharifying tank with 90 L of water, and then, pH was adjusted to 5.35 with acetic acid. Stirring and heating were performed as follows: 50°C for 60 min, 63°C for 50 min, and 70°C for 30 min. The brewer's grains were washed with 80 L water (80°C) and filtrated at 78°C. Then, these grains were boiled for 90 min, during this process ,11, 31, and 28 g hop were added at 10, 20, and 30 min, respectively, and then precipitated 30 min. The wort was cooled by the heat exchanger and fed into a fermentation tank, set to 18°C and added with 3 L of wort (DAB yeast cultured in wort, the quantitative of yeast is 4.2 × 10^5^/ml). Naturally heated to 20 degrees and sealed the fermentation tank when the sugar content was dropped to 4.0%. After 5 days, the wort was cooled down to 4°C and maintained 1 day. Yeast was drained and fermentation temperature slowly drops to 2°C to save beer.

### 2.4 Standard and beer sample preparation

A total of 50 μl of acetaldehyde, 25 μl of isobutyl alcohol, 40 μl of N‐propanol, 16 μl of ethyl acetate, 100 μl of isoamyl alcohol, 0.5 μl of ethyl hexanoate, 5 μl of isoamyl acetate, 0.5 μl of ethyl octanoate, 60 μl of N‐butylalcohol, and 40 ml of ethyl alcohol were added to a volumetric flask. The total volume was determined to be 1,000 ml with ultrapure water.

A total of 500 ml of beer from the fermentation were moved to a conical flask, and the beer was cooled at 4°C to prevent volatile loss.

### 2.5 HS‐SPME procedure and GC–MS analysis

headspace solid‐phase microextraction procedure and GC–MS analysis were based on Li, Liu, Kun‐Farkas, and Kiss (2015) with some modification. Analysis was performed using an Agilent 6890N capillary gas chromatograph (GC, Agilent Technologies Co., Ltd.), and a Combi PAL autosampler (Supelco Co. Ltd, Commonwealth of Pennsylvania) was assembled with a 65 μm polydimethylsiloxane–divinylbenzene (Supelco Co., Ltd., Commonwealth of Pennsylvania) extraction fiber.

A total of 5 ml of the cooled beer mentioned above were placed in 20 ml glass vials and contained 3 g of NaCl and magnetic stirrers. Then, the vial was tightly capped with butyl rubber stopper finally wrapped with an aluminum cap. A fiber was allowed to puncture the cap seal and exposed in the headspace of the vial for 30 min at 40°C with continuous stirring at 250 rpm. After extraction, the fiber was removed from the sample vial and immediately inserted into the heated injector of the gas chromatograph (250°C) for 5 min desorption time.

A GC system (Agilent 6890 N) with HS‐SPME‐suitable injector, an Agilent 5973 mass spectrometry (MS) detector and a HP‐5 capillary column 30 m × 0.25 mm, with 0.25 μm film thickness (Agilent Technologies Co., Ltd.) and N (3.5 ml/min) as the carrier gas, were used to identify the flavor compounds of beer after the HS‐SPME procedure. The injector temperature was programmed to increase from 40°C (held for 2 min) to 250°C in the rate of 7°C/min. The final temperature of 250°C was held for 3 min.

Mass spectrometry was performed in an electron impact ion source mode at 70 eV electron beam, and the scanning range was 35–350 μm. The test voltage, pressure, and transfer line temperature were 0.8 kV, 49.5 kPa, and 250°C, respectively.

### 2.6 Quantitative analysis of volatile compounds

Firstly, the quantitative calibration factor *f_i_* was calculated according to Equation (1), as follows:

(1) $$f_{i}=\frac{A_{s}\times W_{i}}{A_{i}\times W_{s}}$$

where *A_s_* is the peak area of the internal standard, *W_i_* is the identified concentration of a particular standard, *A_i_* is the peak area of particular standard, and *W_s_* is the concentration of the internal standard. Headspace chromatographic profiles were compared with the known reference flavor standard compounds for identification (Table 1).

**Table 1:** Quantitation calibration factors (f_i_) of flavor composition

Volatile compound	Peak area						Concentration (mg/L)	quantitative calibration factor *f_i_*
	1	2	3	4	5	Average		
Acetaldehyde	21.70	21.00	21.20	21.90	21.10	21.40	15.60	0.86
N‐Propanol	6.40	6.60	6.00	6.30	6.80	6.37	32.14	5.96
Ethyl acetate	113.80	110.20	114.20	115.80	108.90	112.97	14.4	0.15
Isobutyl alcohol	25.60	26.40	26.10	26.50	24.80	25.80	20.05	0.92
N‐butanol	58.10	59.00	58.70	56.70	56.80	57.40	48.59	1.00
Isoamyl alcohol	82.30	84.30	77.20	86.10	79.40	80.90	81.00	1.18
Isoamyl acetate	52.90	55.50	52.10	55.10	51.10	52.77	4.38	0.10
Ethyl hexanoate	2.80	2.90	2.60	2.80	2.80	2.73	0.44	0.19
Ethyl octanoate	6.30	6.70	6.20	6.30	6.90	6.47	0.43	0.08

Volatile compounds were determined and quantified with a flame ionization detector, and signals were stored and integrated by a computer software. The concentration of each volatile component (*C_i_*) was calculated using Equation (2), as follows:

(2) $$C_{i}=\frac{A_{i}\times C_{s}\times f_{i}}{A_{s}}$$

where *C_s_* is the internal standard concentration.

### 2.7 Determination of the functional components of beer

The Folin phenol reagent method (Prior, Wu, & Schaich, 2005) was used to measure the total polyphenols content. Briefly, 0.5 ml of beer sample was mixed with 1.0 ml of Folin phenol reagent and then stood for 5 min, added with 2.0 ml saturated sodium carbonate solution and then placed for 1 hr under 30°C water bath. The absorbance was measured at the wavelength of 747 nm by using a UV‐2102PCS spectrophotometer (Unico Instrument Co., Ltd.) after cooling. Total polyphenols content was calculated using chlorogenic acid equivalent (GAE) standard curve and expressed as μg GAE/ml beer.

The total anthocyanins content was determined following method described by (Ivanova et al., 2011) with some modification. Beer was degassed through ultrasonic oscillation method, and 1 g Nylon 66 powder added into 10 ml beer. The sample was oscillated and extracted for 10 min and centrifuged (2000 r/min, 10 min).Discarded the supernatant, washed the precipitation with water, and centrifuged again, repeat three times. The precipitation was dissolved in 12 ml n‐butanol‐hydrochloric (5:1) solution on boiling water bath for 30 min, and the absorbance was measured at the wavelength of 550 nm (1 cm optical path in the cuvette) with a UV‐2102PCS spectrophotometer (Unico instrument Co., Ltd.). The anthocyanins content was calculated using the following equation proposed by Di Stefano & Cravero (1991):

$$\text{Total anthocyanins content}=A_{550\text{nm}}\times 16.7\times d.$$

where *A*_550 nm_ is absorbance at 540 nm, *d* is dilution factor; total anthocyanins content was expressed in mg/L as malvidin‐3‐glucoside equivalents.

### 2.8 Protein component and molecular weight determination

The nitrogenous substance content was measured by Lundin fraction. In acidic solution, high‐molecule nitrogenous substances are easily precipitated by tannin, and high‐molecule nitrogenous substances and medium‐molecule substances are precipitated by phosphomolybdate. Briefly, 25 ml of beer was acidified with 1 ml of H_2_SO_4_ (0.5832 mol/L) and 20 ml of distilled water in 20°C water bath for 20 min, added 16% (16 g tannin:100 g distilled water) tanning solution, constant volume of 50 ml and then filtered immediately. Automatic Kjeldahl Apparatus (k‐370 BUCHI Labortechnik AG) was applied to measure soluble N content (*C_1_*) in filtrate. The acidification of beer was conducted as follows: 25 ml of beer was mixed with sodium molybdate (2.5 ml, 2.43 mol/L) and distilled water (15.0 ml) to incubate in water bath for 20 min at 20°C. After that, 1 ml of H_2_SO_4_ (0.58 mol/L) was added to the resultant mixture and adjusted to 50 ml and filtered for further use. Then, we measured the soluble N content (*C_2_*) in the filtrate. A total of 25 ml of beer total soluble N content (*C_t_*) were measured. High‐molecule nitrogenous substances content: *C_t_*‐*C_1_*; medium‐molecule nitrogenous substance content: *C_1_*‐*C_2_*; low‐molecule nitrogenous substances content: *C_2_*.

A total of 2 ml of beer and 2 ml of acetone were mixed and centrifuged at 8,000 r/min for 10 min. The sediment was solved with 40 μl of distilled water and mixed using a XH‐C vortex mixer (Jintan City Medical Instrument Factory) for 2 min. A total of 20 μl of sample and 20 μl of loading buffer were placed in a 1 ml centrifuge tube and boiled for 3 min.

Protein component molecular weight was analyzed by sodium dodecyl sulfate‐polyacrylamide gel electrophoresis by using a DYY‐8C electrophoresis system (Beijing 61 Biotechnology Co., Ltd.) (Yoon et al., 2010). The 5% stacking and 15% separating gels were used throughout, with a 29:1 (w/w) acrylamide: bisacrylamide ratio. Gelpro32 software was used to treat the decolorized adhesive.

### 2.9 Amino acid analysis

Amino acid constitute and content were analyzed by L‐8900 automatic amino acid analyzer (Hitachi Limited). The chromatographic conditions are as follows: column temperature of 57°C, detection wavelengths of 570 and 440 nm, injection volume of 20 μl, constant flow rate of 0.40 (pump1) and 0.35 ml/min (pump2) for 35 min.

## 3 RESULTS AND DISCUSSIONS

### 3.1 GC‐MS analysis of the flavor compounds with HS‐SPME

**Figure 1:**
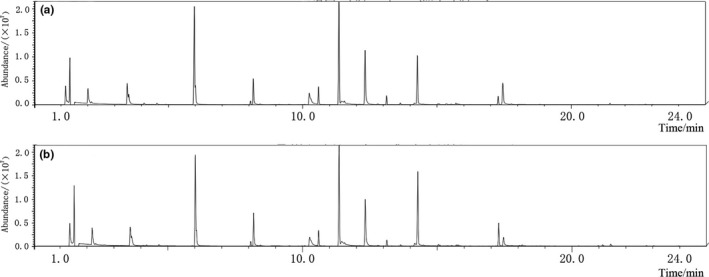
(a) GC–MS analysis of the flavor compounds of extruded black rice adjunct beer. (b) GC–MS analysis of the flavor compounds of rice adjunct beer

The volatile flavor compounds of beer were analyzed by GC‐MS and matched with the experimental spectra that referred to the 1998 data bank 1.6 of the US National Institute of Standards and Technology. The areas of the peaks were calculated manually (Zhang, He, Ma, & Li, 2017). A total of 34 volatile flavor compounds were identified in extruded black rice adjunct beer (Figure 1a and Table 2, matching degree ≥80%), including one organic acid, one aromatic, ten alcohols, and 22 esters. And there are 37 volatile flavor compounds were identified in rice adjunct beer (Figure 1b and Table 2, matching degree ≥80%), including one aromatic, two organic acids, seven alcohols, and 27 esters. All volatile flavor compounds accounted for 89.04% and 86.29% of the total peak area, respectively.

**Table 2:** Volatile compounds of extruded black rice adjunct beer

No.	Compound	CAS	KI	Peak area/%	
				Extruded black rice adjunct beer	Rice adjunct beer
1	Ethanol	64‐17‐5	463	4.29	5.70
2	3‐Methyl‐1‐butanol	123‐51‐3	697	3.24	3.44
3	1‐Propanol	71‐23‐8	562	2.10	–
4	Ethyl Acetate	141‐78‐6	586	3.30	3.00
5	2‐Methyl‐1‐propanol	78‐83‐1	597	1.07	0.28
6	Octanoic acid	124‐07‐2	1,173	1.56	1.88
7	2‐Methylbutylacetat	624‐41‐9	820	2.24	2.29
8	Isobutyl acetate	110‐19‐0	721	0.19	0.14
9	Ethyl butyrate	105‐54‐4	785	0.22	0.23
10	Isoamyl acetate	123‐92‐2	820	17.97	13.87
11	Ethyl hexanoate	123‐66‐0	984	4.52	5.38
12	Phenylethyl alcohol	60‐12‐8	1,136	4.77	3.98
13	Ethyl caprylate	106‐32‐1	1,183	15.86	14.92
14	Octyl acetate	112‐14‐1	1,183	1.65	1.41
15	Phenethyl acetate	103‐45‐7	1,259	10.43	9.08
16	Ethyl nonanoate	123‐29‐5	1,282	0.09	0.12
17	Ethyl trans‐4‐decenoate	76649‐16‐6	1,389	0.26	0.01
18	Ethyl caprate	110‐38‐3	1,381	7.91	12.35
19	3‐Methylbutyl octanoate	2035‐99‐6	1,417	0.14	–
20	2‐Phenylethyl octanoate	5457‐70‐5	1,856	0.09	0.23
21	Dimethyl phthalate	131‐11‐3	1,440	0.22	–
22	1‐Undecanol	112‐42‐5	1,357	0.12	–
23	2,4‐Di‐tert‐butylphenol	96‐76‐4	1,555	0.03	0.02
24	Ethyl laurate	106‐33‐2	1,580	1.31	3.47
25	Diethyl phthalate	84‐66‐2	1,639	4.69	2.26
26	(+)‐Cedrol	77‐53‐2	1,543	0.13	0.05
27	Decanoic acid, 3‐methylbutyl ester	2306‐91‐4	1,615	0.03	0.07
28	Nerolidol	7212‐44‐4	1,564	0.05	–
29	Arachidic acid ethyl ester	18281‐05‐5	2,375	0.02	0.04
30	Geranylgeraniol	24034‐73‐9	2,192	0.03	–
31	Diisobutyl phthalate	84‐69‐5	1,908	0.21	0.34
32	Dibutyl phthalate	84‐74‐2	2,037	0.09	0.07
33	Pentadecanoic acid ethyl ester	41114‐00‐5	1,878	0.04	0.02
34	Geraniol	106‐24‐1	1,861	0.12	–
35	Hexyl acetate	142‐92‐7	984	–	0.16
36	1‐Octanol	111‐87‐5	1,059	–	0.05
37	Enanthylic ether	106‐30‐9	1,083	–	0.03
38	Isopentyl hexanoate	2198‐61‐0	1,218	–	0.04
39	Ethyl dihydrocinnamate	2021‐28‐5	1,359	–	0.02
40	Decanoic acid	334‐48‐5	1,372	–	0.77
41	1‐Dodecanol acetate	112‐66‐3	1,580	–	0.08
42	Phenylethyl octanoate	5457‐70‐5	1,856	–	0.14
43	1‐Dodecanol	112‐53‐8	1,457	–	0.09
44	Phenylethyl caproate	6290‐37‐5	1,657	–	0.26

Note

KI is the retention index calculated by the Kovats method.

Esters, the main carrier of aroma is the dominant flavor compounds in the extruded black rice adjunct beer and constitute an important group of aromatic compounds in beer (Geroyiannaki et al., 2007).The mainly flavor‐active esters in extruded black rice adjunct beer are ethyl acetate which has a fruity or solvent‐like aroma, isoamyl acetate which has a fruity or banana aroma, ethyl caproate, and ethyl caprylate which have a sour apple and fruity aroma, phenethyl acetate which has an apple or rose aroma, and ethyl caprate which has a fruity aroma (Verstrepen et al., 2003). Ethyl ester principal is predominant in extruded black rice adjunct beer. The sum of ethyl esters accounted for >66% of the total esters in beer. Isoamyl acetate accounted for 26% of the total esters which suggested that the threshold value effect on flavor was significant.

The alcohols in beer are mainly higher alcohols, mainly 3‐methyl‐1‐butanol, 1‐propanol, 2‐methyl‐1‐propanol, and phenylethyl alcohol. Higher alcohols are the main components of the fermentation by‐products of beer. These alcohols came from ketonic acid which is related to the synthesis of cellular white matter by yeast. Appropriate amounts of 3‐methyl‐1‐butanol and 1‐propanol gave the beer a feeling of mellowness, and phenethyl alcohol has a pleasant rose aroma (Luigi & Peter, 2008). However, excess higher alcohol gave the beer unpleasant bitter taste and wine essence stimulation effect.

Nerolidol, geraniol, and geranylgeraniol are substances that are undetected in rice adjunct beer. Geranylgeraniol has antitumour, antibacterial, and other physiological activities and maintains cerebral cholesterol (Kotti, Ramirez, Pfeiffer, Huber, & Russell, 2006). Meanwhile, geraniol is an important intermediate in the synthesis of coenzyme Qn (I), vitamin A, vitamin E, and vitamin K_2_, and it is an important precursor for terpenoid synthesis paclitaxel and steroids in organisms. The content of nerolidol and geraniol which may contribute to the aroma by synergized Linalool and citronellol were low.

Ethanol, acetaldehyde, ethyl butanoate, ethyl 2‐methylpropanoate and ethyl 4‐methylpentanoate showed the highest odor activity values (Kishimoto, Noba, Yako, Kobayashi, & Watanabe, 2018). The 2‐phenylethyl octanoate, geraniol, arachidic acid ethyl ester, and pentadecanoic acid ethyl ester contents were low. However, the presence of different esters can have a synergistic effect on the individual flavors which indicated that esters can also affect beer flavor well below their individual threshold concentrations.

### 3.2 Volatile component concentrations

**Figure 2:**
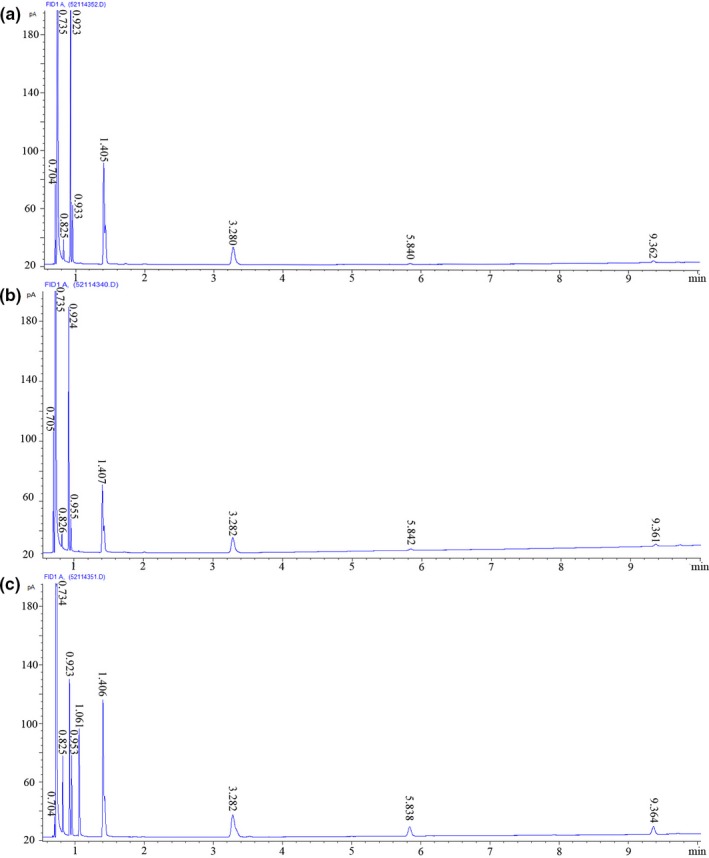
(a) The GC of the extruded black rice adjunct beer. The corresponding compounds and their retention times for the peaks are acetaldehyde (0.704 min), ethanol (0.735 min), N‐propanol (0.825 min), ethyl acetate (0.923 min), isobutyl alcohol (0.953 min), isoamyl alcohol (1.405 min), isoamyl acetate (3.280 min), ethyl hexanoate (5.840 min), and ethyl octanoate (9.362 min). (b) The GC of the rice adjunct beer. The corresponding compounds and their retention times for the peaks are acetaldehyde (0.705 min), ethanol (0.735 min), N‐propanol (0.826 min), ethyl acetate (0.924 min), isobutyl alcohol (0.955 min), isoamyl alcohol (1.407 min), isoamyl acetate (3.228 min), ethyl hexanoate (5.842 min), and ethyl octanoate (9.361 min). (c) The GC chromatogram of the mixed stand sample. The peaks correspond to acetaldehyde (0.704 min), ethanol (0.734 min), N‐propanol (0.825 min), ethyl acetate (0.923 min), isobutyl alcohol (0.953 min), N‐butanol (1.061 min), isoamyl alcohol (1.406 min), isoamyl acetate (3.282 min), ethyl hexanoate (5.838 min), and ethyl octanoate (9.364 min)

Nine flavor compounds, namely acetaldehyde, N‐propanol, ethyl acetate, isobutyl alcohol, N‐butanol, isoamyl alcohol, isoamyl acetate, ethyl hexanoate, and ethyl octanoate, present in extruded black rice adjunct and rice adjunct beers (Figure 2a,b) were identified by detecting the standard solution (Figure 2c). The analyzes were measured in triplicate. The recovery ratios of volatile compounds from the beers ranged from 95.3% to 99.7%. The volatile composition and contents of the extruded black rice adjunct beer were obtained.

Volatile compounds increase the complex flavor more than nonvolatile compounds. Acetaldehyde was the only aldehyde identified among the major volatile compounds, and increased acetaldehyde provides a pungent odor to the beverage and it also leads to health hazards (Geroyiannaki et al., 2007). The acetaldehyde content (14.16 mg/L in extruded black rice adjunct beer and 13.52 mg/L in rice adjunct beer) was lower than those of several kinds of beer on the market (Ceccaroni et al., 2019; Tian, 2010). At low concentrations, the sensory of acetaldehyde is described as “classic,” “nutty,” and “sherry‐like” (Dragone, Mussatto, Oliveira, & Teixeira, 2009).

The ester content in the extruded rice adjunct beer (ethylacetate, isoamyl acetate, ethyl hexanoate, and ethyl octanoate at 12.60, 3.85, 0.40, and 0.37 mg/L, respectively) was higher than that in rice adjunct beer (Table 3). Esters are the main carrier of aroma (Geroyiannaki et al., 2007) and responsible for the fruity character (Verstrepen et al., 2003).

**Table 3:** Concentrations of the volatile beer components

N‐Butyl alcohol		Volatile compound	Extruded black rice adjunct beer		Rice adjunct beer	
Average peak area	Content (mg/L)		Average peak area	Content (mg/L)	Average peak area	Content (mg/L)
53.20	48.59	Acetaldehyde	18.00	14.16	17.20	13.52
		N‐Propanol	5.20	28.32	5.80	32.45
		Ethyl acetate	91.60	12.60	78.40	9.86
		Isobutyl alcohol	20.10	16.85	15.60	12.56
		Isoamyl alcohol	65.10	70.33	56.50	61.55
		Isoamyl acetate	43.00	3.85	42.10	3.60
		Ethyl hexanoate	2.30	0.40	3.10	0.75
		Ethyl octanoate	5.20	0.37	5.00	0.30

The higher alcohols which are a source of fragrance in beer are the by‐products of fermentation process, although it has additional side effects (Strubelt, Deters, Pentz, Siegers, & Younes, 1999). Beer has a stale feeling when it is higher alcohol content >50 mg/L. Isoamyl alcohol was the most abundant alcohol in both samples which accounted for more than two‐thirds of the total alcohol (Table 3), and it is the main cause of drunkenness (Luigi & Peter, 2008). The N‐propanol and isobutyl alcohol contents were low in both beers.

### 3.3 Functional components of beer

The polyphenol content was 79.98 μg GAE/ml (Table 4). The total anthocyanin content and antioxidant activity were positively correlated (Yang, Wei, Mengjie, & Guixing, 2010). Oladokun et al. (2016) analyzed the polyphenols of 34 commercial lager beers, and their results showed that these beers were produced using a range of different raw materials which result in the polyphenol content ranging from 3.91 mg/L to 21.17 mg/L.

**Table 4:** The contents of total polyphenols and anthocyanin in beer

Compound	Light absorption value				Content
	1	2	3	Average	
Polyphenols	0.801	0.803	0.803	0.803	79.98 μg GAE/ml
Anthocyanins	0.129	0.128	0.128	0.128	23.80 mg/L

The main anthocyanins were cyperus japonicas‐3‐O‐glucoside (C3G) and paeoniflorin‐3‐0‐glucoside (P3G). Direct sunlight will damage the structure of anthocyanins. Under acidic conditions (pH of 4–7), anthocyanins were stable for light scattering and thermal stability. The fermentation process was carried out in a low‐temperature (2°C) and light‐tight tank to obtain beer with high anthocyanin content.

Furthermore, some polyphenols should be able to effectively crosslink proteins to form a stable network causing nonbiological precipitation (Siebert, 2006). The basic mechanism underlying the interaction between proteins and polyphenols is that a polyphenol molecule with at least two binding sites attaches to two proteins, and then, bridges are formed between the polyphenol and two proteins to yield a three‐membered structure. The precipitation was insignificant in the extruded black rice adjunct beer which indicated that the interaction between proteins and polyphenols was related to the polyphenol content and the polyphenol types (e.g., monophenolic compounds, nonflavonoid polyphenols, flavonoids, and condensed tannins) (Callemien & Collin, 2010), protein concentrations, polyphenols, polysaccharides, alcohol content, oxygen, pH, temperature, ionic strength, and the presence of metal ions(Siebert, Troukhanova, & Lynn, 1996).

### 3.4 Soluble nitrogen and protein component analysis

The total soluble nitrogen content in extruded black adjunct beer (3,220.54 mg/L) was lower than the wort (4,750.7 mg/L), and the high‐molecule, medium‐molecule, and low‐molecular N contents in extruded black rice adjunct beer were 632.13, 770.38, and 1,817.6 mg/L, respectively (Table 5). The soluble nitrogen provided a nitrogen source for yeast in fermentation, which has been reported in previous research (Silva et al., 2008). The high‐molecule nitrogenous substances ratio is 19.63% in beer, which mainly includes nucleic, proteins and their degradation products, medium‐molecule nitrogenous substances (23.92%) mainly includes polypeptides and protein, and low‐molecule nitrogenous substances (56.45%) mainly includes amino acids and biogenic amine. Nitrogenous compounds are considered extremely important in beer because it affects flavor, foam stability, haze formation, color, yeast nutrition, and biological stability (Marta Fontana, 2009).

**Table 5:** The soluble nitrogen content of extruded black rice adjunct beer

	Total nitrogenous substances	High‐molecule nitrogenous substances	Medium‐molecule nitrogenous substances	Low‐molecule nitrogenous substances
Wort				
Content (mg/L)	4,750.7	1,085.1	100.24	2,663.2
Proportion (%)		22.84	21.10	56.06
Extruded black rice adjunct beer				
Content (mg/L)	3,220.5	632.13	770.38	1817.90
Proportion (%)		19.63	23.92	56.45

**Figure 3:**
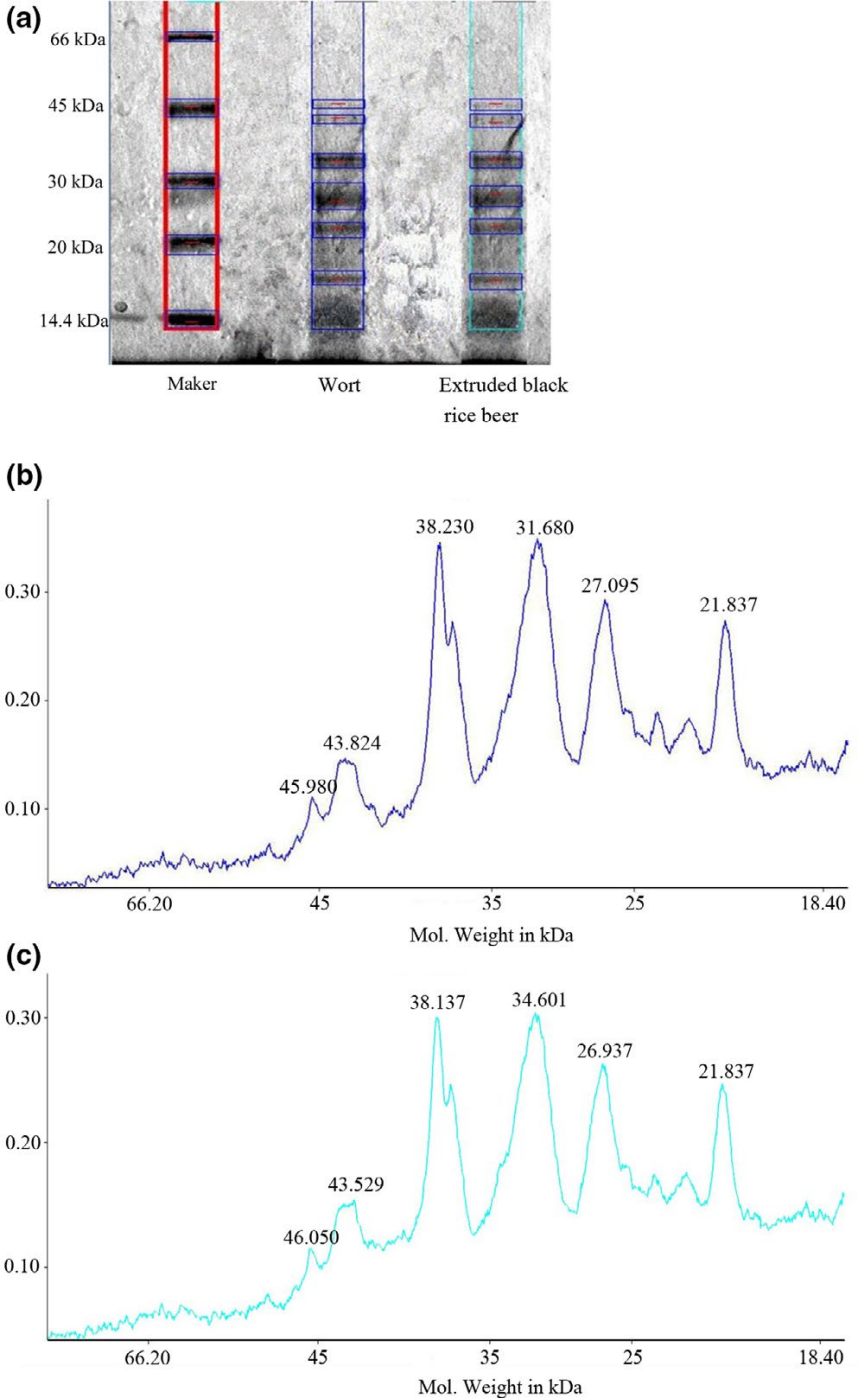
(a) The bands after decoloration. (b) The distribution interval of protein in wort. (c) The distribution interval of protein in extruded black adjunct beer

The relative molecular weight distribution of protein in extruded black rice adjunct beer is shown in Figure 3. The relative protein and polypeptide molecular masses in extruded black rice beer were mainly found in the following five ranges: 20.0–22.0, 25.0–28.5, 30.0–32.0, 37.0–39.0, and 43.0–46.0 kDa. The range of relative molecular weight distribution of protein in wort was same with the beer, but total content was higher. This result was consistent with those of a previous research (Hejgaard & Kaersgaard, 2013). Protein which is as a kind of substance accounting for a large content in beer is the main skeleton component of beer foam. Protein has a considerable influence on beer foam quality. The water‐soluble protein in the raw material is the main source of beer foam protein. Low protein surface hydrophobic property stabilizes beer foam. Beer contains approximately 500 mg/L of proteinaceous material (Hejgaard & Kaersgaard, 2013), typically with the size ranging from 5 kDa to 100 kDa.

Hordein (mainly 10–35 kDa) which is the main protein in barely (40%–50%) that is weakly soluble in water are responsible for haze formation in beer. Protein Z is a main protein with a molecular mass of 43–46 kDa which has high surface viscosity and hydrophobicity. Protein Z is the most important protein to maintain foam stability. Between 20 and 22 kDa barley dimeric α‐amylase inhibitor‐1 (BDAI‐1) is the major protein. Within this range, high protein content was detected in >50 samples in the previous research (Takashi et al., 2008). Lipid transfer proteins (LTPs, 7–14 kDa) are the most abundant proteins in beer foam. LTPs contribute to foam quality by reducing the adverse lipid to stabilize beer foam. Proteins, such as protein Z, LTP‐1, hordein, and BDAI‐1, determine foam stability (Takashi et al., 2008).

### 3.5 Amino acid analysis

**Figure 4:**
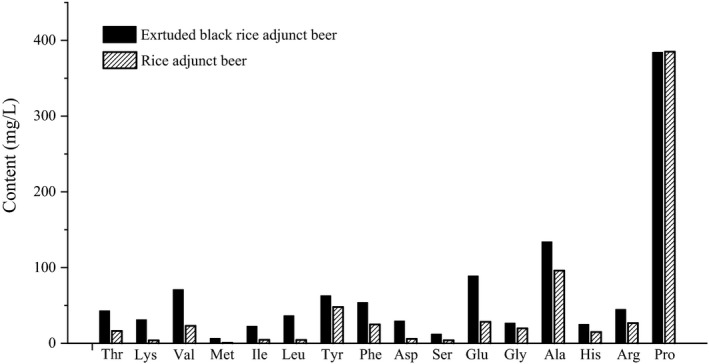
The contents of various kinds of amino acids in beer

Amino acid analyzer method was used to determine amino acid composition and content. Amino acids are one of the important components in beer. Figure 4 shows that 16 kinds of amino acids were detected in extruded black rice adjunct beer and rice adjunct beer. Amino acids have two main sources in beer, that is, one is the action of cereal proteolytic enzymes to release amino acids from the proteins of malt black rice and hops (Poveda, 2019), and the other is secreted into fermentation fluid by yeast metabolism. Amino acids from both of these sources were existed in beer in the state of dissolution (Redruello et al., 2017). There are 15 kinds of amino acids in extruded black rice adjunct beer are higher than rice adjunct beer (Figure 4).

The most abundant amino acids in extruded black rice adjunct beer were Pro, which constituted the majority of the samples (35.8% of the total amino acid content). This finding was consistent with those of previous works (Pomilio, Duchowicz, Giraudo, & Castro, 2010; Poveda, 2019). High amino acid content was caused by the fact that yeasts do not metabolize Pro (Lucie, Thibault, Isabelle, Sylvie, & Carole, 2012). The next most abundant amino acids in extruded black rice adjunct beer were Ala (134.0 mg/L), Glu (88.9 mg/L), Val (70.9 mg/L), Tyr (62.8 mg/L), Phe (52.8 mg/L), Arg(44.7 mg/L), Thr (42.8 mg/L), Leu (36.4 mg/L), and Lys (31.1 mg/L). These results were consistent with the findings of previous studies (Poveda, 2019; Redruello et al., 2017). The contents of the two essential amino acids (Val and Thr) were greater than rice adjunct beer (Figure 4) and other beers (pale dark lager or ale). Compared with different varieties of rice (Liyanaarachchi, Mahanama, Somasiri, & Punyasiri, 2018), the essential amino acid (EAA) content was high in black rice. This phenomenon may be reason why the EAA content was higher in extruded black rice adjunct beer. The sample with the EAA proportion close to 40% in the total free amino acids (FAA) was supposed to good quality (Liu, Wang, & Zhou, 2007). The EAA/FAA of extruded black rice adjunct beer was 35.8%, and high Pro content resulted in low EAA/FAA ratio; meanwhile, the EAA/FAA of rice adjunct beer was 11.1%.

The amino acid content is a fingerprint for the authenticity of beers and can be used as a specific biomarker to identify the end product. Hence, the nature and relative amount of the amino acids can be related to the wort composition and beer fermentation conditions (Pomilio et al., 2010).

## 4 CONCLUSION

Headspace solid‐phase microextraction coupled with gas chromatography–mass spectrometry was used to identify the flavor compounds. A total of 34 volatile flavor compounds were identified. Nerolidol, geraniol, and geranylgeraniol were used the characterize flavor substances compared with rice adjunct and extruded rice adjunct beer. The polyphenol content which influences the antioxidant ability and foam stability was 79.98 mg/L. The composition and content which contribute to nutrition and foam stability were consistent with those of the previous work. A total of 16 amino acids, including 7 EAAs, were identified and quantified. The valine and threonine contents were 70.9 and 42.8 mg/L, respectively.

## CONFLICT OF INTEREST

The authors declared that they have no conflicts of interest to this work. Written informed consent was obtained from all study participants.

## ETHICAL APPROVAL

This study does not involve any human or animal testing.
